# Electrical Properties of Cement-Based Composites with Carbon Nanotubes, Graphene, and Graphite Nanofibers

**DOI:** 10.3390/s17051064

**Published:** 2017-05-08

**Authors:** Doo-Yeol Yoo, Ilhwan You, Seung-Jung Lee

**Affiliations:** 1 Department of Architectural Engineering, Hanyang University, 222 Wangsimni-ro, Seongdong-gu, Seoul 04763, Korea; dyyoo@hanyang.ac.kr; 2 School of Civil, Environmental and Architectural Engineering, Korea University, 145 Anam-ro, Seongbuk-gu, Seoul 02841, Korea; ih-you@korea.ac.kr; 3 Future Strategy Center, Korea Railroad Research Institute, 176 Cheoldobangmulgwan-ro, Uiwang-si, Gyeonggi-do 16105, Korea

**Keywords:** cement composites, nanomaterials, electrical resistivity, gauge factor, self-sensing capacity

## Abstract

This study was conducted to evaluate the effect of the carbon-based nanomaterial type on the electrical properties of cement paste. Three different nanomaterials, multi-walled carbon nanotubes (MWCNTs), graphite nanofibers (GNFs), and graphene (G), were incorporated into the cement paste at a volume fraction of 1%. The self-sensing capacity of the cement composites was also investigated by comparing the compressive stress/strain behaviors by evaluating the fractional change of resistivity (FCR). The electrical resistivity of the plain cement paste was slightly reduced by adding 1 vol % GNFs and G, whereas a significant decrease of the resistivity was achieved by adding 1 vol % MWCNTs. At an identical volume fraction of 1%, the composites with MWCNTs provided the best self-sensing capacity with insignificant noise, followed by the composites containing GNFs and G. Therefore, the addition of MWCNTs was considered to be the most effective to improve the self-sensing capacity of the cement paste. Finally, the composites with 1 vol % MWCNTs exhibited a gauge factor of 113.2, which is much higher than commercially available strain gauges.

## 1. Introduction

In recent years, structural health monitoring (SHM) of reinforced concrete (RC) structures has attracted attention from researchers and engineers [1,2]. The main purpose of performing SHM is generally to monitor the actual stress or strain state as well as detect any damage in the structures during use. There are several types of sensors currently available for the SHM of RC structures, including electric strain gauges (ESGs), fiber Bragg grating (FBG) sensors, and lead zirconate titanate (PZT)-based piezoelectric sensors [2]. The FBG sensor was first introduced in 1978 by Hill et al. [3]. Due to its several advantages compared to conventional gauges, such as its high resistance to electromagnetic interference (EMI), long-term stability, light weight, and small size, intensive studies primarily aimed at measuring mechanical strains and temperatures have been performed since 1989. The PZT-based piezoelectric sensor was first introduced in the early 1990s [4] and it has generally been applied for damage detection in structures based on impedance. However, local investigation of the strain, temperature, and damage of RC structures is only possible when the abovementioned artificial sensors, i.e., ESG, FBG, and PZT, are adopted, because they are locally embedded or attached to the structures. This limits the SHM of entire structural elements and the sensors need to be applied intentionally by predicting the weakest parts in the structures. Improper analysis of the determination of the weakest parts in the structures will thus make the application of the sensors for SHM useless.

In order to overcome the drawbacks of the sensors mentioned above, some researchers [5,6,7,8,9,10,11,12,13,14,15,16] have attempted to develop cement-based piezoresistive sensors. The main concept of cement-based sensors is to produce cement composites as a conductive material, and the stress or strain state in the composites can be predicted by measuring the changes in electrical resistivity. Since this novel sensor is based on cement composites, there is no heterogeneity when it is embedded in concrete structures. Furthermore, the SHM of entire structural elements made of concrete is possible by incorporating carbon-based nanomaterials into the concrete mixture, thus conferring conductivity and self-sensing capacity. Banthia et al. [9] investigated the electrical resistivity of cement composites with carbon and steel fibers. A significant decrease of the resistivity was achieved by incorporating both carbon and steel fibers, but carbon fibers were more effective in improving the conductivity compared to steel fibers. D’Alessandro et al. [12] also examined the self-sensing capacity of cement composites with carbon nanotubes (CNTs) for SHM applications. They [12] stated that a sonication procedure is not suitable for large scale applications and as a result, several types of dispersants were adopted to achieve a good dispersion of CNTs without sonication. The percolation threshold of composites including CNTs with and without sonication was suggested to be approximately 1% by weight of cement (1 wt %) and the gauge factors of the paste, mortar, and concrete were proposed to be 130, 68, and 23, respectively. A mathematical model of the electrical conductivity of cement composites with CNTs was also proposed by García-Macías et al. [15,16] based on a micromechanics approach. In their numerical studies [15,16], two important findings were obtained: (1) an increase of the CNT’s aspect ratio leads to a decrease of the percolation threshold and (2) the wavy state and agglomeration of CNTs are the dominant factors for determining the conductivity of cement composites. Le et al. [13] investigated the applicability of two-dimensional graphene nanoplatelets in cement composites for damage sensing. In their study [13], the percolation threshold was found to be about 2.4 vol % and mathematical equations were suggested to correlate the fractional change of resistance with the fractional change of elastic compliance in a mode-I load, based on linear elastic fracture mechanics. Pisello et al. [14] also recently examined the effects of various types of nanomaterials (at 2% by mass of cement) on several properties of cement-based composites, including thermal characteristics, electrical properties, strain-sensing capacities, etc. From a number of test results, they reported that the use of graphene nanoplatelets is most effective in increasing the thermal conductivity and diffusivity, while the addition of CNTs is most effective in improving the strain sensing capacity [14]. Likewise, to date, several studies have been performed for the source technology development of cement-based piezoresistive sensors. However, most previous studies have evaluated the electrical or self-sensing capacities of cement composites including a single type of nanomaterial or ordinary carbon-based materials such as steel or carbon fibers. On the other hand, to the best of the authors’ knowledge, there are limited published studies [14] investigating the comparative self-sensing capacities of cement composites with various recently developed nanomaterials such as multi-walled carbon nanotubes (MWCNTs), graphene (G), and graphite nanofibers (GNFs). Accordingly, in this study, the effect of the nanomaterial type on the electrical properties of cement paste was examined. For this, three different types of nanomaterials—MWCNTs, G, and GNFs—were incorporated into the paste at an identical volume fraction of 1%. The feasibility of producing conductive cement-based composites and to monitor cyclic compressive stresses was investigated. A gauge factor corresponding to the sensing sensitivity of the composites with MWCNTs was also suggested.

## 2. Test Program

### 2.1. Materials, Mix Proportions, and Preparation of Specimens

Table 1 shows the detailed mixture proportions used in this study. As cementitious materials, Type 1 ordinary Portland cement and silica fume (SF) were incorporated. The physical properties and chemical compositions of the cementitious materials used are summarized in Table 2. The reason for including SF in the mixture is that the use of SF decreases the amount and size of large pores in hydrated cement paste and the continuity level of conductive pathways in the composites can be improved, as recently reported by Kim et al. [11]. The water-to-cementitious material (W/CM) ratio employed was 0.35 and the ratio of SF to cement was 0.3. In order to achieve a conductive property in the cement paste, 1% (by volume) of the three different types of carbon-based nanomaterials, MWCNTs, GNFs, and G, were incorporated. In order to achieve good dispersion of the nanomaterials, a sonication process was applied for all test series using a Q500 sonicator at a frequency of 20 kHz. In addition, the dispersion of nanomaterials may be influenced by the fluidity of the cement paste, and thus, the flow values obtained in all test series were controlled to be 150 ± 10 mm by controlling the amount of superplasticizer (SP). The data was evaluated from the flow table test according to ASTM C1437 [17].

**Figure 1:**
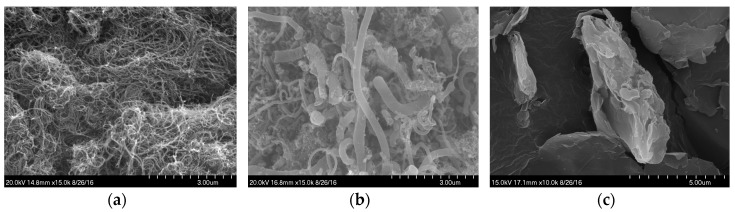
SEM images for nanomaterials: (**a**) MWCNT, (**b**) GNF, (**c**) G.

In order to investigate the morphology of MWCNTs, GNFs, and G, scanning electron microscopy (SEM) images were obtained through a microscopy and imaging facility (S-4700, Hitachi Ltd., Tokyo, Japan). Each material investigated was placed on the holder with attached carbon tapes. Before the observation, the sample was coated by using Hitachi E-1030 Ion Sputter. The dimensions and physical properties of the carbon-based nanomaterials used are summarized in Table 3, and the SEM images for the nanomaterials are shown in Figure 1. The MWCNTs, GNFs and G used in this study were obtained from Carbon Nano-material Technology Co., Ltd. (Pohang, Korea). Since the intrinsic properties which were provided from the above corporation were limited, the strength properties of the nanomaterials were not included in Table 3.

**Figure 2:**
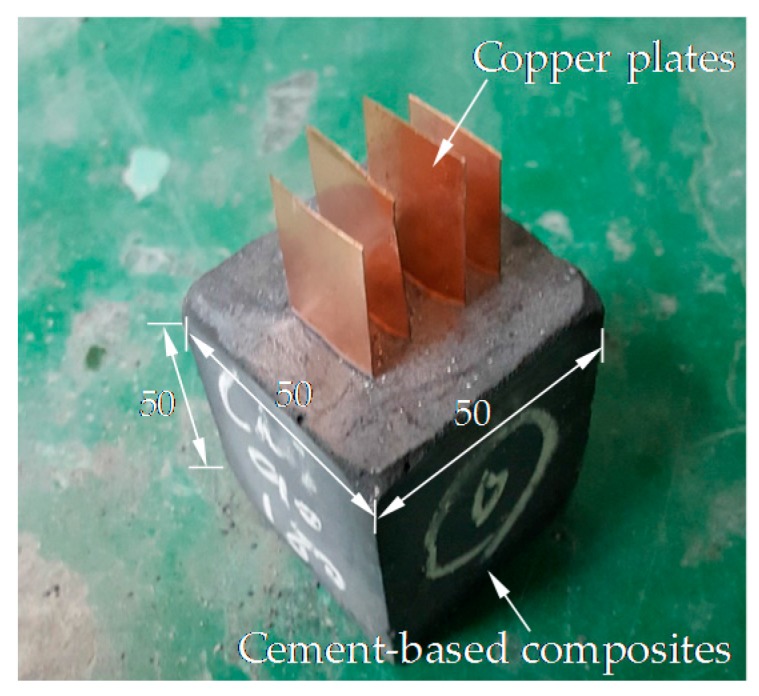
Cubic specimens (unit: mm).

As shown in Figure 1, the MWCNTs and GNFs have cylindrical shapes with aspect ratios of 667 and higher than 50, respectively, while G has a thin plate shape and was randomly aggregated. To evaluate the effects of the nanomaterial type on the electrical properties of the cement paste, cyclic and monotonic compressive tests were performed. For this analysis, cubic specimens with a cross-section of 50 × 50 mm^2^ and a height of 50 mm were fabricated, as shown in Figure 2. In order to achieve good compactness, all cubic specimens were vibrated on a vibration table and then cured in a chamber at a temperature of 23 ± 1 °C and a relative humidity of 60 ± 5% for 28 days (just before testing). A four-electrode method was used in this study to measure the resistance. As shown in Figure 2, four copper plates with a width of 20 mm and a height of 75 mm were embedded into the cubic specimen immediately after casting of cement paste. During hydration process of cement with water, hydration products formed a complex with copper in the plate’s surface and copper ions in solution, and as a result, a strong bond between the copper plates and cement paste was achieved [18]. The embedment length and interval between the copper plates were thus to be obtained as 50 mm and 10 mm, respectively.

### 2.2. Test Setups

To evaluate the electrical properties of the cement composites containing MWCNTs, GNFs, and G, the electrical resistivity was measured as a function of age. The electrical resistivity can be calculated from the following equation: *ρ* = *R* × *A*/*l*, where *R* is the resistance, *A* is the cross-sectional area of the composite in contact with the electrode, and *l* is the space between the two voltage poles. This means that to obtain the resistivity, the electrical resistance, *R*, needs to be measured first. To measure the resistance in the composites, a 819 LCR meter (GWINTEK) was used. In accordance with Chen et al. [19], the resistance increased with time when a direct current was applied due to the polarization effect. Thus, an alternating current at a frequency of 100 kHz was adopted to eliminate polarization problems in this study.

The two probe method can compose simpler circuit compared to four probe method. However, the four probe method was more widely used to ignore contact resistance between electrodes [20,21]. Thus, in this research, the four probe method was employed. In the four probe method, the resistance was measured according to the voltage of the inner electrodes and the flow of current of the two outer electrodes. Two outer and inner electrodes were connected to alternating current, voltage terminals of LCR meter using coaxial cables of 3 m, respectively. To evaluate self-sensing properties of cement-based sensors, the LCR meter and a computer were linked with RS232C interface for automatic data acquisition.

**Figure 3:**
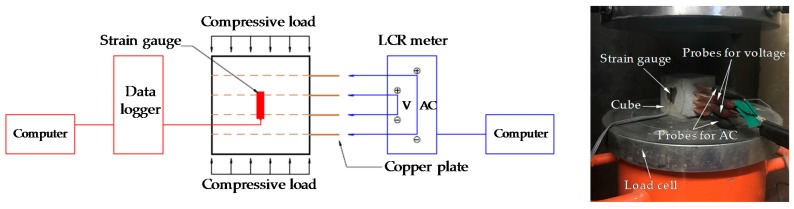
Experimental setup for compressive tests.

**Figure 4:**
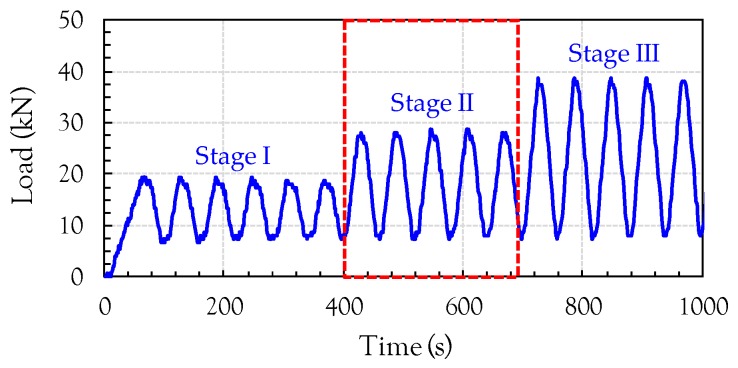
Loading protocol for cyclic compression.

The resistivity of all cubic specimens was measured at 3, 7, 14, 21, and 28 days without any compressive load. After 28 days, compressive loading tests were performed. The detailed test setup for the compressive tests is shown in Figure 3. In order to compare the electrical resistivity and compressive stress/strain of the composites, the load was measured from a load cell affixed to the cross-head of a universal testing machine with a maximum capacity of 250 ton. In addition, the strain was measured utilizing 10 mm long strain gauges attached to the side surfaces of the cubic specimens. Both the strain and load data were acquired using a static data logger. The variation of resistance while changing the load was simultaneously measured using the LCR meter. For the case of cyclic compression, the loading rate, defined as *LR* = ± 0.333 × *n* (kN/s), where *LR* is the loading rate and *n* is the loading phase, was changed in every phase. The applied loading protocol for cyclic compression is given in Figure 4. However, for the case of monotonic compression, to evaluate the gauge factor, a uniaxial load was applied to the cubic specimens at a rate of 0.1 mm/s.

## 3. Test Results and Discussion

### 3.1. Electrical Resistivity of Cement Composites Containing Various Nanomaterials Considering the Curing Age

**Figure 5:**
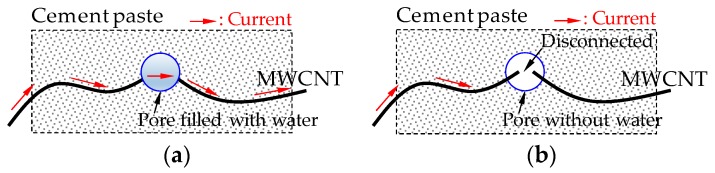
Schematic description for conductive pathway: (**a**) full saturation condition, (**b**) dry condition.

It is well-known that the electrical resistivity of cement-based composites varies with age due to changes of the amount of pore water [9,10]. The electrical resistivity of the composites including conductive nanomaterials also changes as the curing age increases if the amount of nanomaterials included is below the percolation threshold. This is because the pore water helps to establish a continuous conductive pathway for current flow [11]. For instance, as the pore is filled with water, a continuous conductive pathway is established, as shown in Figure 5a, whereas if the water in the pore evaporates, the conductive path becomes disconnected, as shown in Figure 5b. Therefore, the electrical resistivity of plain cement paste and cement composites with an insufficient amount of carbon-based nanomaterials varies with the curing age.

**Figure 6:**
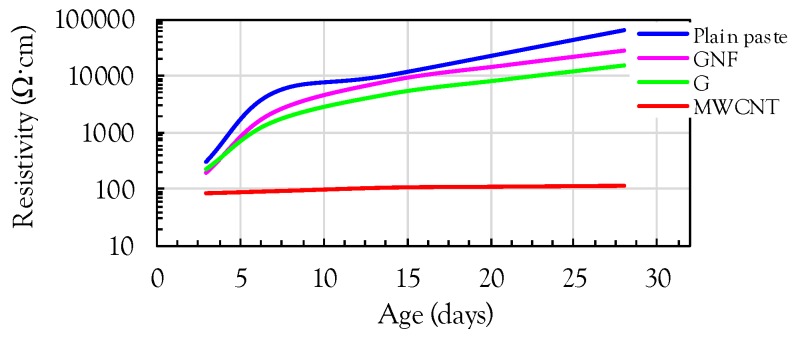
Effect of nanomaterial on electrical resistivity of cement paste with ages.

In order to verify the above explanation, the effects of the nanomaterial type and curing age on the electrical resistivity of the composites were investigated, as shown in Figure 6. It is obvious that the electrical resistivity continuously increased with increasing curing age for the case of the plain cement paste and cement paste with GNFs and G, whereas only a minor change of the resistivity was observed for the cement paste with MWCNTs. This indicates that although identical amounts of GNFs, G, and MWCNTs were incorporated (1 vol %), only the composite with MWCNTs was close to or beyond the percolation threshold value. As the amount of carbon-based nanomaterials is higher than the percolation threshold value, it can be assumed that the continuous conductive pathways are well established. As a result, since conductive pathways formed by the connection of nanomaterials are continuously established, the electrical resistivity is insignificantly influenced by the amount of water in pores. According to a previous study by Kim et al. [6], the percolation threshold for MWCNTs was between 0.3% and 0.6% based on the weight of cement and if the density of MWCNTs is assumed to be 1.2 g/cm^3^, the corresponding percolation threshold values are 0.35 vol % and 0.71 vol %, respectively. This is less than the amount of MWCNTs (1 vol %) used in this study. Therefore, the variation of the electrical resistivity in the composite with MWCNTs with the curing age was relatively insignificant even though a slight increase of the resistivity was observed. This is consistent with the findings of Chen et al. [19] where a minor change of the resistivity with age was obtained when the amount of carbon-based fibers was beyond the percolation threshold value.

The composites with GNFs and G exhibited slightly lower electrical resistivities than the plain paste. However, the effectiveness of reducing the resistivity was insignificant compared to the case of MWCNTs. Similar to the case of plain paste, the electrical resistivities of the composites with GNFs and G increased with age because the amounts of GNFs and G included were insufficient to produce continuous conductive pathways. A slightly higher electrical resistivity was obtained for the case of GNFs at late ages compared to G. This may be because due to the cylindrical shape of GNFs, more pores were formed in the cement paste containing GNFs compared to G, which was randomly aggregated with two-dimensional thin plates. Gong et al. [22] reported that the addition of graphene oxide effectively refines the microstructure by decreasing the amount of capillary pores by 27.7% and the total porosity by 13.5%, owing to its two-dimensional shape.

**Figure 7:**
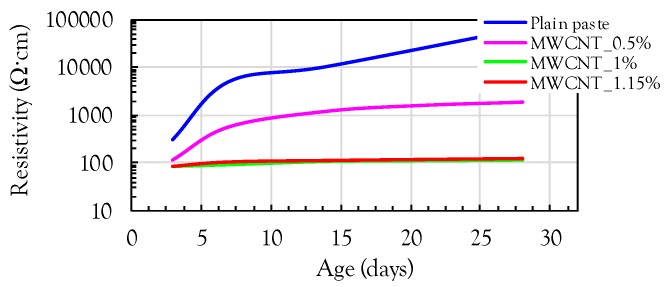
Effect of amount of MWCNT on electrical resistivity of cement paste with ages.

In order to examine the effect of the amount of MWCNTs on the electrical resistivity of the cement paste, additional specimens were fabricated with different volume fractions (0.5 and 1.15 vol %) of MWCNTs. Figure 7 shows the comparisons of the electrical resistivities of the cement pastes with and without MWCNTs. It was obvious that a significant reduction of the resistivity occurred by including MWCNTs, regardless of age. The composites with more than 1 vol % MWCNTs exhibited much smaller resistivities than those with 0.5 vol % MWCNTs. This indicates that the addition of 0.5 vol % MWCNTs is insufficient to produce conductive cement composites. In addition, the composites with 1 and 1.15 vol % MWCNTs exhibited very similar values of resistivity at all ages, demonstrating that 1 vol % MWCNTs can be roughly considered as the percolation threshold. This value is slightly lower than the value suggested by D’Alessandro et al. [12] of 1 wt % (=1.15 vol %), but higher than those suggested by Kim et al. [6] of 0.3–0.6 wt % (=0.35–0.70 vol %).

### 3.2. Piezoresistive Sensing Capacity of Cement Paste with Various Nanomaterials

To evaluate the piezoresistive sensing capacity of cement-based composites with nanomaterials, the fractional change of the resistance was determined as follows:

Δ*R*/*R*_0_ = (*R_x_* − *R*_0_)/*R*_0_ (1)

where *R*_0_ is the initial resistance of the cement-based composites and *R_x_* is the resistance of the cement-based composites under a compressive load. If the dimensional changes of the specimens are negligible during loading, the fractional change of the resistivity, referred to as the *FCR* (Δ*ρ*/*ρ*_0_, where *ρ*_0_ is the initial resistivity), is consistent with the fractional change of the resistance. Thus, for simplification, Equation (1) was used to investigate the self-sensing capacity of the composites.

**Figure 8:**
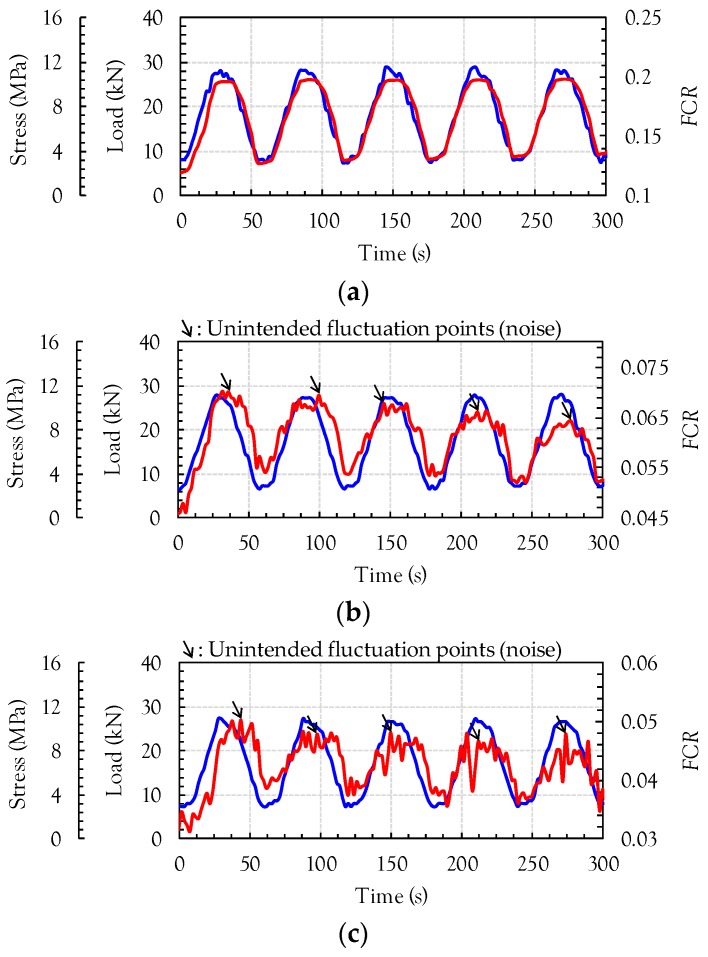
Comparative cyclic compressive behaviors of composites with (**a**) MWCNT, (**b**) GNF, (**c**) G.

**Figure 9:**
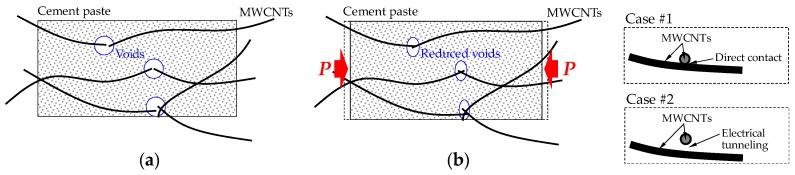
Schematic description of MWCNT link in composites: (**a**) without load; (**b**) with compressive load.

Figure 8 shows the comparison between the compressive load (or compressive stress) and FCR of the cement-based composites incorporating 1 vol % of the three nanomaterials, MWCNTs, GNFs, and G, under cyclic loads. Since unstable data was obtained for both the load and electrical resistance at the initial loading stage, only Stage II in Figure 4 was utilized for the data analysis. Thus, the starting point of Stage II was considered as a zeroing point in Figure 8. The electrical resistivity decreased with increasing compressive load because the nanomaterials become closer, leading to an increase of the conductive pathways. Thus, negative values of FCR were obtained in this study. To directly compare it to the compressive load, the calculated FCR was multiplied by a value of −1. It was obvious that the composites with MWCNTs exhibited the best self-sensing capacity for a cyclic compressive load and a very smooth FCR curve was obtained without any noise, as shown in Figure 8a. This is consistent with the recent findings by Pisello et al. [14]. This is because the amount of MWCNTs used in this study was higher than the percolation threshold values (0.3–0.6 wt %) suggested by Kim et al. [6]. The composites with MWCNTs also provided the highest FCR values at similar compressive loads between 10 and 30 kN. For example, the highest FCR was found to be 0.2, which is approximately 2.9 and 4.0 times higher than the FCR values of the composites with GNFs and G, respectively. The FCR curve showed a strong relationship with the compressive load, where it increased or decreased with increasing or decreasing applied load, respectively. This is because the shape of MWCNTs and distances between MWCNTs changed due to the external load, as reported by Wen and Chung [5]. In particular, the distances between MWCNTs may become closer under a compressive load, causing a lower electrical resistivity by forming more conductive pathways, as shown in Figure 9. The conductive pathways can be formed by connecting the MWCNTs directly (Case #1). In addition, although the MWCNTs are not directly in contact with each other, the electrons can be transferred due to a tunneling effect when they become closer from the external compressive load (Case #2), as reported by García-Macías et al. [15,16]. The cut-off distance between MWCNTs for the tunneling effect was suggested to be 0.5 nm [23]. Consequently, the decrease of distances between MWCNTs resulted in the reduced resistivity, leading to the variations of FCR. A full reversibility was achieved without any residual FCR. The average compressive strength of the plain cement paste was found to be 35.2 MPa and the maximum stress applied to the prismatic specimens was approximately 12 MPa, which is 34% of the compressive strength. In accordance with a previous study [24], micro-cracks start to be formed in the cement paste at nearly 50–60% of the peak strength. Thus, it can be assumed that the prims tested under cyclic compression exhibit no micro-cracks and are within the elastic range. Consequently, full reversibility of the FCR in the composites with 1 vol % nanomaterials was observed.

The cement composites including 1 vol % GNFs and G also exhibited similar behaviors with the applied cyclic compressive loads, as shown in Figure 8b,c. However, there was obvious noise with much smaller FCR values compared to the case of MWCNTs, even though an identical volume fraction (1%) was applied. This means that a poorer self-sensing capacity of the cement composites was obtained by incorporating GNFs and G instead of MWCNTs. It is well known that high signal noise is generally obtained when a poor connectivity of carbon nanomaterials is obtained [25]. The conductive pathways in the composites with GNFs and G were insufficiently formed to flow electrical current, and as a result, the high signal noises were obtained. The aspect ratio of GNFs was much smaller than that of the MWCNTs (Table 3). Thus, since they had similar lengths of approximately 0.01 mm, the number of MWCNTs included was much higher than that of GNFs at the identical volume fraction. Accordingly, the continuous conductive pathway was more effectively created in the composites with MWCNTs due to connections of individual nanotubes compared to the case of GNFs. In the case of G, the most noticeable noise in the FCR was observed because it was composed of randomly aggregated, thin, and crumpled sheets, as shown in Figure 1c. Therefore, compared to cylindrical-shaped nanomaterials (MWCNTs and GNFs), a continuous conductive pathway was hardly formed in the composites containing G even at an identical volume fraction. According to the test results reported by Wen and Chung [7], the composites with 1 vol % carbon fibers also noticeably exhibited electrical noise. Synthetically, it can be concluded that the use of MWCNTs is most effective in improving the self-sensing capacity of cement paste under a cyclic compressive load, compared to those of GNFs, G, and carbon fibers.

### 3.3. Correlation between the Cyclic Compressive Stress and FCR in the Composites with Nanomaterials

**Figure 10:**
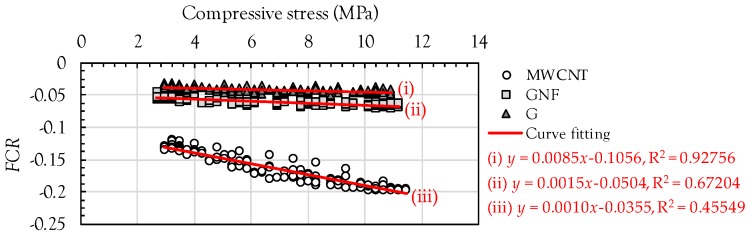
Relationship between cyclic compressive stress and FCR.

Figure 10 shows the relationship between the compressive stress and FCR in the composites with MWCNTs, GNFs, and G. Since all of the specimens were within the elastic range, an almost linear relationship was observed. In order to quantitatively evaluate the sensitivity of the composites to the load, the following linear equation was simply adopted: *y* = −*ax* − *b*, where *a* and *b* are the slope and *y*-axis intercept, respectively. The data deviation and coefficients were strongly influenced by the nanomaterial type. The composites with MWCNTs exhibited the lowest data deviation, corresponding to the highest value of the coefficient of determination (R^2^ = 0.92756), and gave the highest coefficient of *a* = 0.0085, denoting the slope of the relationship. The coefficient *a* is closely related to the sensitivity of sensors, and as a result, the composites with MWCNTs were considered to be most highly sensitive to the cyclic compressive load. On the other hand, the composites with GNFs and G provided much smaller values of R^2^ and *a* compared to the composites with MWCNTs. In particular, the specimens including G exhibited the lowest values of R^2^ and *a*, indicating that they were least sensitive to the compressive load because of the shape of G. Since G is composed of randomly aggregated thin sheets, a continuous conductive pathway was not effectively formed as compared to the MWCNTs and GNFs. Sun et al. [8] also experimentally verified that an insufficient conductive network was obtained in the cement composites with 1 vol % graphite nano-platelets because this volume fraction is lower than the percolation threshold. Therefore, at the identical volume fraction of 1%, the order of sensitivity to the cyclic compressive load was determined as follows: MWCNT > GNF > G.

### 3.4. Gauge Factor

Based on previous test results, it is obvious that composites including MWCNTs are most feasible to be used as a piezoresistive sensor. In addition, the composites with GNFs and G exhibited totally different behaviors between FCR-time and strain-time curves, due to the insufficient amounts of GNFs and G incorporated. Therefore, it was determined that only the composites with MWCNTs are appropriate for a cement-based piezoresitive sensor, and thus, they were only used to analyze the gauge factor. The feasibility of the composites containing MWCNTs under compressive behavior can be quantitatively evaluated based on the gauge factor, as follows:

(2) $$GF\;=\;\frac{\Delta \rho /\rho_{0}}{\varepsilon }\;=\;\frac{FCR}{\varepsilon }$$

**Figure 11:**
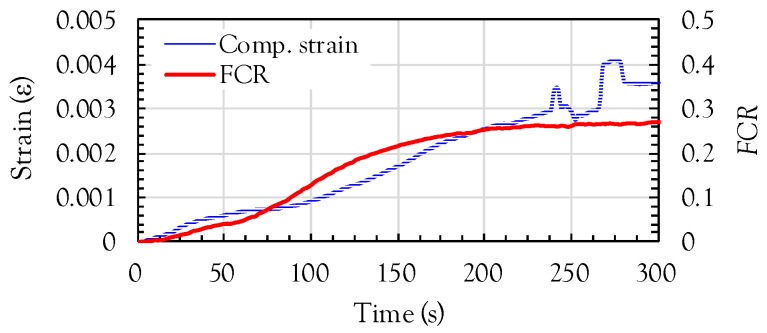
Comparison of compressive strain-time and FCR-time curves for composites with MWCNT.

Here, *GF* is the gauge factor and *ε* is the compressive strain. To obtain the relationship between the FCR and compressive strain in the composites containing MWCNTs, a monotonic compressive test was additionally performed and the compressive strain- and FCR-time curves are shown in Figure 11. Under a compressive load, a lower electrical resistivity was obtained due to the connection of nanotubes, and as a result, a negative FCR was obtained. However, to directly compare the FCR with the strain, the FCR obtained from the experiments was also multiplied by −1. Increases of both the strain and FCR were observed with increasing applied compressive load. Once the compressive strain value reached almost 0.003, unstable strain data was obtained due to the formation of surface cracks in the cubic specimens. Thus, it was assumed that the compressive strain measured from the attached strain gauge was only valid from the origin (zero strain) to a value of 0.003. A slightly higher nonlinearity was found for the case of FCR compared to the strain. The reduction of resistivity by forming more conductive pathways under compression is not linearly related to the increasing load. This is caused by the fact that: (i) the nanotubes are randomly dispersed and oriented in the cement matrix and (ii) the microcracks generated in the matrix change the shape of nanotubes and their connectivity.

**Figure 12:**
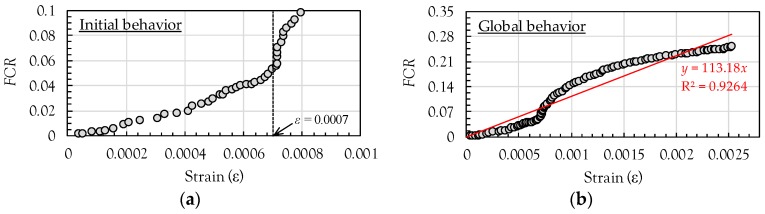
Compressive strain versus FCR relationships of composites with MWCNT: (**a**) initial behavior; (**b**) global behavior.

According to a previous study performed by Han and Ou [26], an almost linear relationship between the FCR and compressive strain up to nearly 600 με (0.0006) was obtained. This is consistent with the findings of this study, as shown in Figure 12a. An almost linear relationship between the FCR and strain was found up to nearly 0.0007. However, the nonlinearity in the relationship became more pronounced with increasing strain (above ε = 0.0007), mainly caused by a steep increase of FCR after this point. From this observation, it is inferred that the degree of formation of conductive pathways in the composites with MWCNTs under compression is dependent on the magnitude of the load applied. However, since the point giving the significant nonlinear behavior between FCR and strain might be varied by several factors, such as degree of MWCNT’s dispersion, pore size distribution, amount of MWCNTs, etc., a further study is required to be done to provide more reasonable explanation. The global behavior between the FCR and strain is shown in Figure 12b before reaching the point where unstable strain data started to be obtained. Based on this global behavior, the gauge factor was calculated by a simple linear regression analysis. The gauge factor was found to be 113.2 and the coefficient of determination (R^2^) was 0.9264. Copper-nickel or nickel-chrome alloy-based strain gauges have a gauge factor of about 2 [27], which is much smaller than that of the cement composites with 1 vol % MWCNTs. In addition, the gauge factor obtained in this study is quite similar to the value obtained in previous studies [12]. D’Alessandro et al. [12] reported that the gauge factor of the cement composites with 1 wt % cement CNTs was found to be 130. Consequently, the cement composites with 1 vol % MWCNTs can be used as a piezoresitive sensor for evaluating compressive behaviors of cement paste, mortar, or concrete.

## 4. Conclusions

This study investigated the effects of nanomaterial type on the electrical properties of cement paste. The electrical resistivity was first estimated at various ages and the cyclic compressive stress was then simulated with the FCR. Finally, the gauge factor was evaluated by comparing the FCR with the strain under a monotonic compressive load. From the above discussions, the following conclusions may be drawn:

(1) The electrical resistivity in the plain cement paste and cement composites with GNFs and G obviously increased with age. In contrast, the composites with MWCNTs exhibited only a minor change of resistivity with age. The order of the conductivity was as follows: MWCNT > G > GNF > plain paste.
(2) At the identical volume fraction of 1%, the composites with MWCNTs provided the best self-sensing capacities under cyclic compression including a higher value of FCR and minor noise, followed by those with GNFs and G.
(3) Based on the compressive test results, the MWCNTs were demonstrated as a proper nanomaterial for cement-based piezoresistive sensors and the gauge factor of the composites including 1 vol % MWCNTs was found to be 113.2.

## Figures and Tables

**Table 1:** Mixture proportions.

	W/CM *	Unit Weight (kg/m^3^)					
		Water	Cement	Silica Fume	MWCNT	GNF	G
Plain mortar	0.35	708	1416	607	-	-	-
w/MWCNT	0.35	708	1416	607	16	-	-
w/GNF	0.35	708	1416	607	-	26	-
w/G	0.35	708	1416	607	-	-	27

Superplasticizer was incorporated by 1 wt % of cementitious materials (cement + silica fume). * W/CM = water-to-cementitious material ratio.

**Table 2:** Chemical composition and physical properties of the cementitious materials.

Composition % (mass)	Cement	Silica Fume
CaO	61.33	0.38
Al_2_O_3_	6.40	0.25
SiO_2_	21.01	96.00
Fe_2_O_3_	3.12	0.12
MgO	3.02	0.10
SO_3_	2.30	-
Specific surface area (cm^2^/g)	3413	200,000
Density (g/cm^3^)	3.15	2.10
Ig. loss (%)	1.40	1.50

**Table 3:** Properties of nanomaterials.

	*d_f_* (nm)	*L_f_* (mm)	*T* (mm)	Layer	Carbon Content (%)	*L_f_*/*d_f_*	Density (g/cm^3^)
MWCNT	15	0.01	3.4–7	-	>90	667	1.20
GNF	200	0.01–0.03	-	-	>90	>50	1.94
G	-	0.01	3–6	3–10	>99	-	2.21

MWCNT = multi-walled carbon nanotube, GNF = graphite nanofiber, and G = graphene, *d_f_* = diameter, *L_f_* = length, *T* = thickness, and *L_f_*/*d_f_* = aspect ratio.
